# A randomised clinical trial of intrapartum fetal monitoring with computer analysis and alerts versus previously available monitoring

**DOI:** 10.1186/1471-2393-10-71

**Published:** 2010-10-28

**Authors:** Diogo Ayres-de-Campos, Austin Ugwumadu, Philip Banfield, Pauline Lynch, Pina Amin, David Horwell, Antonia Costa, Cristina Santos, João Bernardes, Karl Rosen

**Affiliations:** 1 Departmento de Ginecologia e Obstetrícia, Faculdade de Medicina do Porto, Alameda Hernani Monteiro 4200-319 Porto, Portugal; 2 Department of Obstetrics and Gynaecology, St. George's Hospital, University of London, Blackshaw Rd, London, SW17 0QT, UK; 3 Department of Obstetrics and Gynaecology, Glan Clwyd Hospital, Rhyl, Denbighshire, LL18 5UJ, Wales, UK; 4 Department of Obstetrics and Gynaecology, Ninewells Hospital, Dundee, DD1 9SY, Scotland, UK; 5 Department of Obstetrics and Gynaecology, University Hospital of Wales, Heath Park, Cardiff, CF14 4XW, Wales, UK; 6 Department of Obstetrics and Gynaecology, Luton and Dunstable Hospital, Lewsey Road, Luton, Bedfordshire LU4 0DZ, UK; 7 Departmento de Ginecologia e Obstetrícia, Faculdade de Medicina do Porto, Alameda Hernani Monteiro 4200-319 Porto, Portugal; 8 Serviço de Bioestatística e Informática Médica, Faculdade de Medicina do Porto, Alameda Hernani Monteiro 4200-319 Porto, Portugal; 9 Departmento de Ginecologia e Obstetrícia, Faculdade de Medicina do Porto, Alameda Hernani Monteiro 4200-319 Porto, Portugal; 10 Department of Engineering, University of Borås, Allégat 1, SE-501 90 Borås, Sweden

## Abstract

**Background:**

Intrapartum fetal hypoxia remains an important cause of death and permanent handicap and in a significant proportion of cases there is evidence of suboptimal care related to fetal surveillance. Cardiotocographic (CTG) monitoring remains the basis of intrapartum surveillance, but its interpretation by healthcare professionals lacks reproducibility and the technology has not been shown to improve clinically important outcomes. The addition of fetal electrocardiogram analysis has increased the potential to avoid adverse outcomes, but CTG interpretation remains its main weakness. A program for computerised analysis of intrapartum fetal signals, incorporating real-time alerts for healthcare professionals, has recently been developed. There is a need to determine whether this technology can result in better perinatal outcomes.

**Methods/design:**

This is a multicentre randomised clinical trial. Inclusion criteria are: women aged ≥ 16 years, able to provide written informed consent, singleton pregnancies ≥ 36 weeks, cephalic presentation, no known major fetal malformations, in labour but excluding active second stage, planned for continuous CTG monitoring, and no known contra-indication for vaginal delivery. Eligible women will be randomised using a computer-generated randomisation sequence to one of the two arms: continuous computer analysis of fetal monitoring signals with real-time alerts (intervention arm) or continuous CTG monitoring as previously performed (control arm). Electrocardiographic monitoring and fetal scalp blood sampling will be available in both arms. The primary outcome measure is the incidence of fetal metabolic acidosis (umbilical artery pH < 7.05, BD_ecf _> 12 mmol/L). Secondary outcome measures are: caesarean section and instrumental vaginal delivery rates, use of fetal blood sampling, 5-minute Apgar score < 7, neonatal intensive care unit admission, moderate and severe neonatal encephalopathy with a marker of hypoxia, perinatal death, rate of internal monitoring, tracing quality, and signal loss. Analysis will follow an intention to treat principle. Incidences of primary and secondary outcomes will be compared between groups. Assuming a reduction in metabolic acidosis from 2.8% to 1.8%, using a two-sided test with alpha = 0.05, power = 0.80, and 10% loss to follow-up, 8133 women need to be randomised.

**Discussion:**

This study will provide evidence of the impact of intrapartum monitoring with computer analysis and real-time alerts on the incidence of adverse perinatal outcomes, intrapartum interventions and signal quality. (Current controlled trials ISRCTN42314164)

## Background

Intrapartum complications accounted for 512 perinatal deaths in the UK in 2004 [1] and remain an important cause of long-term neurological morbidity and suffering for families in industrialised countries [2]. In more than half of such cases there is evidence of suboptimal care, where different management would reasonably have been expected to have made a difference to the outcome [3]. Problems related to fetal surveillance are the most commonly reported in these cases, and cardiotocograph (CTG) interpretation is the basis for the most frequent criticism [3].

The aim of intrapartum monitoring is to identify fetuses at risk of death or long-term injury caused by decreased oxygen supply during labour. The majority of cases of death and long-term disability are caused by situations other than poor oxygenation during labour, so to establish this diagnosis it is necessary to document the occurrence of relevant changes in umbilical blood gas values after birth. Umbilical artery metabolic acidosis has been associated with an increased risk of neurological injury [4], and is commonly used as a proxy measure for adverse clinical outcome in this setting.

CTG monitoring remains the basis of intrapartum fetal surveillance in high-risk cases and is applied on a wide scale in industrialised countries, but its interpretation by health professionals has a well documented poor reproducibility [5,6], and the technology has not been shown to improve the most important clinical outcomes, but rather to increase operative delivery rates [7]. Fetal blood sampling (FBS) can be used in addition to CTG, but it is invasive and time consuming, and only provides time-limited information, all of which have limited its application on a wide scale [8].

The addition of fetal electrocardiogram ST waveform analysis to conventional CTG (STAN^®^, Neoventa, Gothenburg, Sweden) has been shown to increase the identification of fetuses with metabolic acidosis [9]. A systematic review of the first three trials comparing CTG+ST monitoring with conventional CTG showed that the former significantly decreases the rates of fetal blood sampling, neonatal encephalopathy and operative delivery, and is associated with a borderline reduction in the incidence of umbilical artery metabolic acidosis [10]. It has recently been documented that adverse neonatal outcomes continue to occur with routine use of the STAN^®^ technology, because of human errors, such as poor CTG interpretation, delay in taking appropriate action, or failure to follow clinical guidelines [11], as well as non-occurrence or very late occurrence of ST events [12]. There is now a consensus among STAN^®^ users that visual interpretation of the CTG remains the main weakness of the technology [13].

The Omniview-SisPorto^®^ 3.5 program (Speculum, Lisbon, Portugal) provides computer analysis of both CTG and ST signals, incorporating the concept of centralised viewing of tracings on multiple stations and real-time alerts for healthcare professionals [14]. The system has been shown to provide analysis of CTG events that is in good agreement with a consensus of experts [15] and the program's alerts have been shown to be highly predictive of fetuses born with severe acidemia [16], so it has the potential to overcome some of the weaknesses associated with human interpretation of the CTG. There is now a need to determine whether the use of this technology will result in improved perinatal outcomes.

## Aims

The primary aim of the study is to determine whether computer analysis of intrapartum fetal monitoring signals with real-time alerts (Omniview-SisPorto^®^ 3.5) will reduce the rate of umbilical artery metabolic acidosis compared to continuous electronic fetal monitoring as previously performed. Secondary aims are to quantify other measures of perinatal outcome, intervention rates and signal quality measures in both arms of the study.

The rationale for the main hypothesis of the study is that real-time alerts are expected to prompt healthcare professionals to identify, and act on changes that would otherwise remain unnoticed. The technology may also reduced human errors associated with inappropriate CTG interpretation, including lack of identification of cases showing reduced fetal heart rate variability [12]. Observational data suggest that the validity of computerised intrapartum fetal monitoring provides additional advantages [16].

Written informed consent for enrolment will be requested from all participants. The trial is registered at Current Controlled Trials with the number ISRCTN42314164. The study protocol was approved by the Cambridgeshire 1 Research Ethics Committee (REC reference number 09/H0304/61)

## Methods/design

### Study Design and Setting

This is a pragmatic multicentre randomised clinical trial, to be carried out in five United Kingdom hospitals, including three tertiary teaching units and two district general hospitals, all with high-risk women in labour.

### Population and Methods

#### Participants/Eligibility criteria

Women will be eligible for participation if they fulfil the following criteria:

• singleton pregnancy with cephalic presentation

• gestation ≥ 36 completed weeks

• no known major fetal malformations

• in active labour but not in active second stage

• no known contra-indication to vaginal delivery

• clinical decision made to perform continuous CTG monitoring

Patients will not be included if they are under 16 years of age, or are not able to provide written informed consent.

#### Dissemination of the study to potential participants

Patient study information (posters and leaflets) will be distributed to eligible women on their initial contact with healthcare professionals, and revisited during antenatal and parentcraft classes, which take place at different stages of pregnancy. The same information will also be available to women on arrival in labour at participating hospitals.

#### Consent request and enrolment

Eligible women will be asked by their attending midwife whether they wish to participate when the clinical decision is made to perform continuous fetal monitoring during labour. The indication for instituting monitoring will be recorded. If receptive, they will be asked for written informed consent and subsequently enrolled in the trial, in a window that is automatically opened by the Omniview-SisPorto^®^ 3.5 program. If enrolment does not take place, the occurrence of exclusion criteria or the reasons reported by women for not participating will be registered.

#### Randomisation

After enrolment, women will be randomised to one of two arms using a 1:1 computer-generated randomisation sequence attributed to each centre by the Omniview-SisPorto^®^ 3.5 program.

#### Intervention arm

Women randomised to the intervention arm will have continuous fetal monitoring during labour with computer analysis by the Omniview-SisPorto^®^ 3.5 program, with real-time alerts to changes detected on CTG and/or ST signals [14]. In this arm, ultimate management decisions remain the responsibility of healthcare professionals, according to their best clinical judgment. This may include CTG+ST analysis and/or FBS. However, non-directive guidelines are provided to help understand the meaning of the various alerts (Table 1).

**Table 1:** Non-directive guidelines used to help understand the meaning of the various alerts

**Signal loss/maternal heart rate monitoring**	Consider re-positioning the Doppler probe, changing to internal FHR monitoring, re-evaluating the scalp electrode connections, or changing the electrode
**ST signal loss**	Consider re-evaluating the scalp electrode connections or changing the electrode
**Tachysystole **(excessive number of uterine contractions)	Consider discontinuing/reducing oxytocin infusion or acute tocolysis
**Yellow alerts **(tracing characteristics that do not fulfil the criteria of normality, but are not usually associated with significant fetal hypoxia)	Consider maintaining close monitoring and/or starting ST analysis if available
**Orange alerts **(FHR+ST characteristics that may be associated with some degree of fetal hypoxia)	Consider reversal of hypoxic causes if possible, maintaining close monitoring, starting ST analysis if available, or performing FBS
**Red alerts **(FHR+ST characteristics that are likely to be associated with fetal hypoxia)	Consider immediate reversal of causes of hypoxia if possible or immediate delivery

#### Control arm

Women randomised to the control arm will have continuous fetal monitoring during labour without computer analysis or alerts, and will be managed according to the centre's existing guidelines. This may include ST analysis and/or FBS as adjuncts to standard CTG.

#### Umbilical cord blood analysis

All enrolled cases will be subject to immediate double cord clamping and paired cord blood sampling (umbilical artery and vein), which are prerequisites for accurate diagnosis of metabolic acidosis. Sampling into two pre-heparinised syringes should be delayed by no more than 30 minutes, air bubbles will be removed, the syringes capped, and blood analysis will occur in less than 30 minutes [17].

#### Collection of baseline, labour and outcome data

Baseline, labour and outcome data will be obtained by the local research midwife on the next working day after delivery and entered into the Omniview-SisPorto^®^ 3.5 program. Baseline demographic and obstetric data of enrolled women will include maternal age, height, weight; pre-existing or current medical conditions; third trimester group B streptococcus carrier state if known, number of previous pregnancies; number of previous vaginal, instrumental, and caesarean deliveries. Data on the current labour will include gestational age; spontaneous or induced; normal or augmented; presence of slight or thick meconium staining of amniotic fluid; occurrence of fever (temp ≥38°C); use of epidural, parenteral, or inhaled analgesia; other intrapartum medications; result(s) of FBS; date and time of birth; interval between end of fetal monitoring and delivery; normal, instrumental, or caesarean delivery; indication for instrumental or caesarean delivery. Outcome data will include newborn birthweight, sex; 1 and 5-min Apgar scores; umbilical artery pH (3 decimal places), pCO2, bicarbonate, BE_ecf_; umbilical vein pH (3 decimal places), pCO2, bicarbonate, BE_ecf_; neonatal intensive care unit admission and indication. These data will be transmitted in an anonymised format to the co-ordinating centre. Cord acid base data will be assessed for accuracy. If cord blood samples are not available, neonatal data will be used to asses the occurrence of metabolic acidosis.

#### Late outcome and serious adverse event data collection

Cases with metabolic acidosis (umbilical artery pH < 7.05 and BE_ecf _> 12 mmol/L), 5-min Apgar score < 7, or neonatal intensive care unit admission will be further investigated by the local research midwife to evaluate whether: neonatal blood analysis was performed in the first hour of life, and its results including lactate; neonatal encephalopathy of any grade occurred in the first 72 hours of life; death of the infant occurred in the first 28 days of life; other important neonatal complications occurred in the first 7 days of life; brain ultrasound or other imaging technologies were performed in the first 7 days of life, and their results. At the same time, serious adverse events (see data safety monitoring committee, below) will be sought and reported.

#### Primary outcome measure

The primary outcome measure of this study is the incidence of fetal metabolic acidosis (defined as an umbilical artery pH < 7.05, BD_ecf _> 12 mmol/L). BD_ecf _will be calculated from pH and PCO_2 _values, according to the Siggaard-Andersen acid-base algorithm [18]. Subgroup analysis, by participating centre and by availability or non-availability of ST analysis will be carried out.

#### Secondary outcome measures

The secondary outcomes are: overall rates of caesarean section and of caesarean section for non-reassuring fetal state; overall rates of instrumental vaginal delivery and of instrumental vaginal delivery for non-reassuring fetal state; fetal blood sampling rates; incidence of 5-minute Apgar score < 7; need for neonatal intensive care unit admission; incidence of moderate and severe neonatal encephalopathy with a hypoxic marker; perinatal death; rate of delayed interventions (interval between red alerts - offline analysis in the control arm - and delivery in metabolic acidosis cases); internal FHR monitoring rates; tracing quality and signal loss.

#### Sample size

The sample size calculation is based on the primary endpoint: a metabolic acidosis incidence of about 2.8% can reasonably be assumed, as this was previously reported in an observational study conducted in one of the participating centres [11]. Since this will be the first trial to evaluate the effect of computer analysis of intrapartum fetal monitoring signals and real-time alerts on perinatal outcomes, it is not possible to find an estimate of the degree of change that is expected. The closest available parallel is the evaluation of STAN^®^ monitoring versus conventional CTG during labour. A systematic review of the first three trials that studied this issue revealed an overall relative risk for metabolic acidosis of 0.64. In the absence of a better alternative, this will be the value used for initial sample size calculation. Thus assuming a reduction in metabolic acidosis from 2.8% to 1.8%, with an alpha of 0.05, a two-sided test, and a power of 0.80, about 7320 women will need to be randomised. Accounting for a 10% loss to follow-up, the study requires the inclusion of 8133 women in order to obtain the 7320 analysable cases (3660 per arm). A pilot analysis will be conducted after enrolment of the first 1500 cases to evaluate the real incidence of metabolic acidosis, the effect of real-time alerts on its rate, and the need to recalculate the trial's sample size.

#### Data analysis

Data analysis will be carried out at the co-ordinating centre and analysis of the primary endpoint will follow the intention to treat principle. Minimal differences between groups are expected in baseline patient characteristics. The incidence of metabolic acidosis will be compared across both groups, using relative risk with 95% confidence intervals. A similar methodology will be applied to secondary outcomes.

#### Missing data

A case will be classified as having metabolic acidosis if it falls within the described diagnostic criteria and the arterial sample shows a pH value at least 0.03 units lower and a PCO_2 _value at least 1 kPa higher than the venous sample [18,17]. If these two latter criteria are not met, or only one sample is available, it will be considered as indicating a venous sample. Venous samples will still be considered for the diagnosis of metabolic acidosis if they meet the diagnostic criteria. If no cord blood acid base data is available, the case may still be classified as having metabolic acidosis if the neonate has acid-base data obtained in the first hour of life that fulfil the diagnostic criteria, or if a lactate value greater than 10 mmol/L is documented in association with signs of maladaptation (RDS, need for buffering etc).

### Local Research Co-ordinators and Local Research Midwifes

A research co-ordinator in each centre will be responsible for planning of the trial and for dissemination of information to staff. A local research midwife will contribute to these tasks, and be responsible for dissemination of information to possible participants, encouraging high levels of patient recruitment, regular and timely collection and recording of data, and provision of regular feedback to the co-ordinating centre on study progress and potential protocol violations.

### Ethical Considerations

Written informed consent from all participants will be obtained before trial enrolment. This is the first time that computer analysis of intrapartum fetal monitoring with real-time alerts is being compared with conventional fetal monitoring in a randomised trial, so there is no prior evidence of benefit for the reduction in the incidence of adverse neonatal outcomes. However, there are observational data to suggest that computer analysis of fetal monitoring signals has a higher validity in prediction of adverse outcomes [16].

The confidentiality of personal data is guaranteed by the fact that person-identifiable data will not be available to anyone outside the local healthcare team. All data transmitted to the co-ordinating centre will be anonymised automatically.

### Independent Data Safety Monitoring Committee

All serious adverse events (see below) will be reported to the Data Safety Monitoring Committee, who will evaluate their frequency regularly and determine whether there is a significantly increased incidence in the intervention group and if so, whether the study should be discontinued.

This committee will consist of a neonatologist, an obstetrician, and a statistician. Serious adverse events are defined as any of the following:

• *severe metabolic acidosis (umbilical artery pH < 7.00 and BE_ecf _> 12 mmol/L) and neonatal intensive care unit admission*

• *5-min Apgar score < 7 and neonatal intensive care unit admission*

• *First available pH value after birth < 7.05 or first available lactate value after birth > 10 mmol/L*

• *Grade II or III neonatal encephalopathy*

• *Death in the first 28 days of life*

## Competing interests

Authors DAC, CS and JB are employed by the University of Porto, the main sponsor for this study; this institution receives royalties from the commercialisation of the Omniview-SisPorto^®^ program, and has financed the publication of this manuscript.

## Authors' contributions

All authors made substantial contributions to the conception and design of the study protocol. DAC and AU wrote the first version of the manuscript, which received input from the remaining authors. CS was the main author responsible for the statistical input. All authors read and approved the final version of the manuscript.

## Pre-publication history

The pre-publication history for this paper can be accessed here:

http://www.biomedcentral.com/1471-2393/10/71/prepub
